# Supplementary material for: Enhancing tuberculosis treatment support: A thematic analysis of interactive messages in a digital adherence technology trial to identify needs, challenges, and strategies for improvement
Source: PLoS One. 2025 Jul 24;20(7):e0326492. doi: 10.1371/journal.pone.0326492 (PMC12289081; doi:10.1371/journal.pone.0326492)
Supplement: Attachment — Submitted filename: PLOS ONE COMMENTS.docx [file pone.0326492.s002.docx]

Thank you for the opportunity to review Dr. Sarah J. Iribarren paper entitled “Digital Technology to Support Tuberculosis Treatment: Thematic Analysis of Interactive Messages to Understand Needs, Challenges, and Strategies to Improve Support Tools”. I hope these comments of mine will perhaps help this research.

1. Refrain from using too many abbreviations such as TS, TB-TST, DAT and SM if this will be used in the succeeding texts. Much better to spell it out instead.
2. In the inclusion criteria, it indicated “16 years of age or older” maybe it would be better to specify the maximum age for the criteria. Additionally, better to include people with access to Wi-Fi or mobile data since most of the application requires it to improve the quality of data collection.
3. If the study wants to show the effectiveness of this method, qualitative findings might be rich in details, but it also lacks quantitative validation. Consider a mixed-methods approach that combines qualitative thematic analysis with quantitative metrics (e.g., adherence rates, treatment outcomes especially for long-term) to provide a more comprehensive understanding.
4. It is great that limitations are stated in the research. Maybe it would be better to include potential biases as well.
